# Screw-Type Collar vs. Non-Screw-Type Collar Implants—Comparison of Initial Stability, Soft Tissue Adaptation, and Early Marginal Bone Loss—A Preclinical Study in the Dog

**DOI:** 10.3390/biology11081213

**Published:** 2022-08-12

**Authors:** Haim Tal, Vadim Reiser, Sarit Naishlos, Gal Avishai, Roni Kolerman, Liat Chaushu

**Affiliations:** 1 Department of Periodontology and Oral Implantology, The Maurice and Gabriela Goldschleger School of Dental Medicine, Tel Aviv University, Tel Aviv 6997801, Israel; 2 Department of Oral & Maxillofacial Surgery, The Maurice and Gabriela Goldschleger School of Dental Medicine, Tel Aviv University, Tel Aviv 6997801, Israel; 3 Department of Oral & Maxillofacial Surgery, Rabin Medical Center, Beilinson Campus, Petah Tikva 4941492, Israel; 4 Department of Pedodontics, The Maurice and Gabriela Goldschleger School of Dental Medicine, Tel Aviv University, Tel Aviv 6997801, Israel

**Keywords:** dental implant, marginal bone loss, soft tissue healing, 2nd-stage surgery, implant collar

## Abstract

**Simple Summary:**

Implant neck characteristics may affect initial implant stability, soft tissue healing, and early marginal bone loss (EMBL) at second-stage surgery. Screw-type rough-surface collar implants had statistically significant poorer soft tissue healing and increased marginal bone loss compared to non-screw type implants at the time of 2nd-stage surgery. The significance of the novel implant design results in preventing EMBL awaits further research.

**Abstract:**

Background: Implant neck characteristics may affect initial implant stability, soft tissue healing, and early marginal bone loss (EMBL) at second-stage surgery. The null hypothesis was that, following two-stage implant insertion, rough surface, non-screw-type collar implants will present lower EMBL at 2nd-stage surgery than rough-surface, screw-type collar implants. Methods: The study comprised seven male beagle dogs (mean weight 10.57 ± 2.8 kg; range 9–17 kg). A novel implant design was developed, composed of 2 parts: an apical part resembling a regular threaded implant, and a coronal non-screw-type collar, 4.2 mm long, served as the study group, whereas standard threaded implants served as control. Twenty-eight implants were placed: two on each side of the mandible. All implants were sand-blasted/acid-etched and of similar dimensions. Each dog received four implants. To assess location (anterior vs. posterior) impact on the outcomes, implants were placed as follows: group I—posterior mandible right—non-screw-type collar implants; group II—anterior mandible right—similar non-screw-type collar implants. To assess the collar-design effect on the outcomes, implants were placed as follows—Group III—anterior mandible left—control group, screw-type collar implants; Group IV—study group, posterior mandible left—non-screw-type collar implants. The following parameters were measured and recorded: insertion torque, soft tissue healing, early implant failure, and EMBL at 2nd-stage surgery. Results: No statistically significant differences were noted between groups I and II regarding all outcome parameters. At the same time, although insertion torque (55 N/cm) and early implant failure (0) were similar between groups III and IV, group III presented significantly poorer soft tissue healing (1.43 vs. 0.14) and increased marginal bone loss (0.86 vs. 0 mm). Conclusions: When a two-stage implant protocol was used, rough-surface non-screw-type collar implants led to superior outcomes at 2nd-stage surgery. Implant location did not affect the results. The significance of this result in preventing EMBL awaits further research.

## 1. Introduction

While Bränemark et al. [1] coined the term osseointegration in dentistry (1977), limited early marginal bone loss (EMBL) around dental implants was considered acceptable [2,3]. Nowadays, EMBL is regarded as a potential risk for implant health [4]. EMBL is highly influenced by implant-related factors such as microgeometry [5,6,7].

Originally, submerging implants during the healing period was considered essential for osseointegration [1,8] protecting the implants from external forces, microorganisms, and other oral hazards [7] and, therefore, minimizing the risks associated with the healing process.

The Bränemark implants, as well as other multiple studies using the same screw-type configuration, reported high survival rates [9,10,11,12,13,14]. Notably, similar encouraging results were reported for implants with different modifications in the macro- and microgeometry aiming to promote peri-implant osseointegration [15,16].

The observations of EMBL in the case of two-piece implants seemed to have been unavoidable and constantly appeared soon after implants were exposed or after early spontaneous exposure [17]. Hence, macrostructure and surface characteristics may be an essential component for maintaining soft tissue integrity.

As for now, although changes such as implant neck length and design, diameter, surface characteristics, micro threads, one-piece implants, and platform switching were studied, no evidence for the superiority of any of the new designs or configurations has been agreed upon [18]. Conflicting results exist regarding the optimal neck configuration for early marginal bone preservation. Previous meta-analysis and reviews showed that a rough-surfaced micro thread design in the implant neck may reduce the amount of EMBL compared to a machined surface or a conventional rough surface [19,20,21]. Contradictorily, a recent review failed to provide evidence regarding the influence of implant geometry on EMBL and survival or success rates 1 year following implant placement [22].

Recently, a novel implant composed of two compartments was designed. The implant contains two parts: a. an apical part resembling a regular threaded implant and b. a thread-free removable collar [23]. The purpose of the new two-piece dental implant is to avoid/treat peri-implant contamination, which is a frequent event [23]. The present study aimed to assess the impact of the modification in the implant neck characteristics on the initial implant stability, soft tissue healing, and EMBL at 2nd-stage surgery. The null hypothesis was that rough-surface non-screw-type collar implants may minimize EMBL following surgical or early spontaneous soft tissue exposure compared to rough-surface, screw-type collar implants.

## 2. Materials and Methods

### 2.1. Novel Design of the Dental Implant

**Figure 1:**
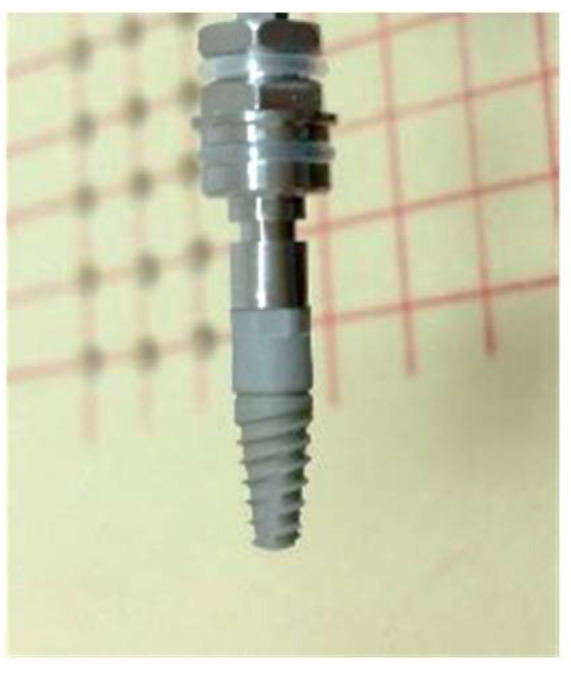
A novel non-screw-type removable collar implant design.

A novel implant (NGI 100™, Implant B, Nazareth, Israel) composed of 2 compartments was designed, consisting of a. an apical part resembling a regular threaded implant and b. a thread-free 4.2 mm-long removable collar (Figure 1) [23].

The fixture is a sand-blasted acid-etched rough-surface implant. Both the implant and the collar were made of identical Ti-6Al-4V ELI (Grade 23 Titanium). The implant’s total length was 10 or 13 mm long and 4.2 mm in diameter. The abutment connection was a standard 2.45 mm internal hexagon.

### 2.2. Implant’s Stability

**Figure 2:**
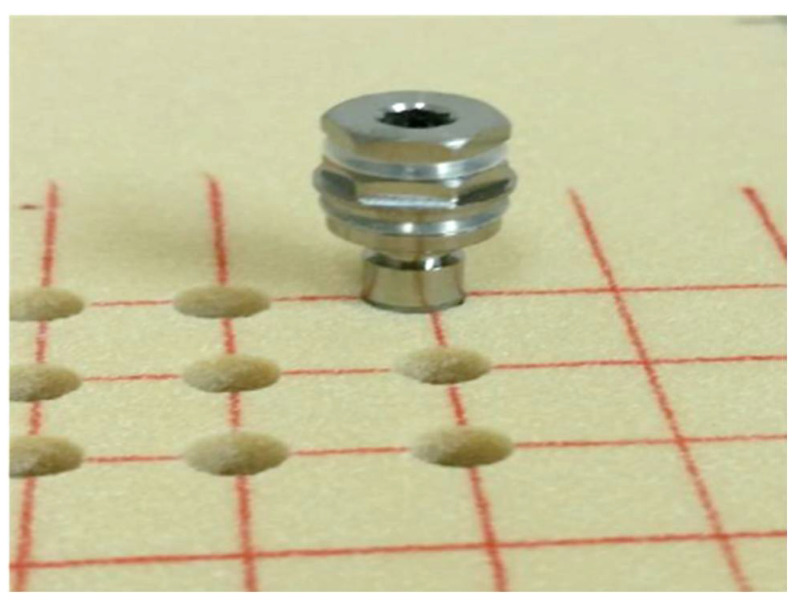
Acceptance criteria: insertion torque ≥50 N/cm; full insertion flush with the bone model surface.

A pilot in vitro study tested implants inserted into rigid polyurethane foam of 30 pounds per cubic foot (PCF), (SAWBONES^®^, Washington, DC, USA) (Figure 2).

**Figure 3:**
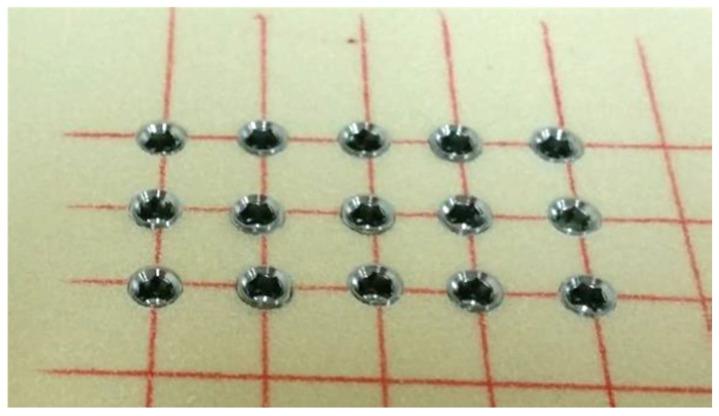
Fifteen implants were examined for any deviation from the acceptance criteria.

The drilling was performed using a standard protocol, including marking the insertion point using a round marking drill, followed by a pilot drill up to the final depth (13 mm) of the osteotomy. This was followed by drilling with 2.8 mm, 3.2 mm, and 3.65 mm drills to the final depth (13 mm) and a 4.2 mm diameter drill up to 6 mm only to allow initial stability from the apical part and avoid unnecessary coronal stress on the crestal alveolar bone. Insertion torque ≥50 N/cm at full implant placement flush with the model surface was accepted as a required criterion for stability. This was achieved by the insertion of the implants into the osteotomy by gradually increasing the insertion torque of the physiodispenser until the implants were flush with the model surface. The final torque was recorded. Fifteen implants were placed using this protocol (Figure 3). After implantation, the insertion element was removed, and the implants were visually examined for any deviation from the expected results.

### 2.3. Experimental Prospective In Vivo Study

#### 2.3.1. Animals

The study comprised seven male beagle dogs (mean weight 10.57 ± 2.8 kg; range 9–17 kg). The age of animals was between 17–20 months. The number of animals was in accordance with current regulations and scientific integrity. The dogs were housed under environmental controlled conditions in the animal facility of the GLP Jesus Usón Minimally Invasive Surgery Center (JUMISC) of Cáceres, Spain. Food consisted of a solid diet. Water was given ad libitum by automatic watering incorporated in each cubicle. The study procedures have been reviewed and approved by the ethical committee of animal experimentation of JUMISC of Caceres, Spain, and authorized by the competent administration of the board of Extremadura, Spain, to ensure it meets the criteria established by the RD 53/2013 (Directive 2010/63/EU of the European Parliament and of the council of 22 September 2010), (Approval No.—20160927-3, 2016). A veterinarian evaluated all dogs once a day to assure their good health and condition. In addition, this manuscript has been written in line with the ARRIVE guidelines for reporting animal research [24].

#### 2.3.2. Implant Distribution

**Figure 4:**
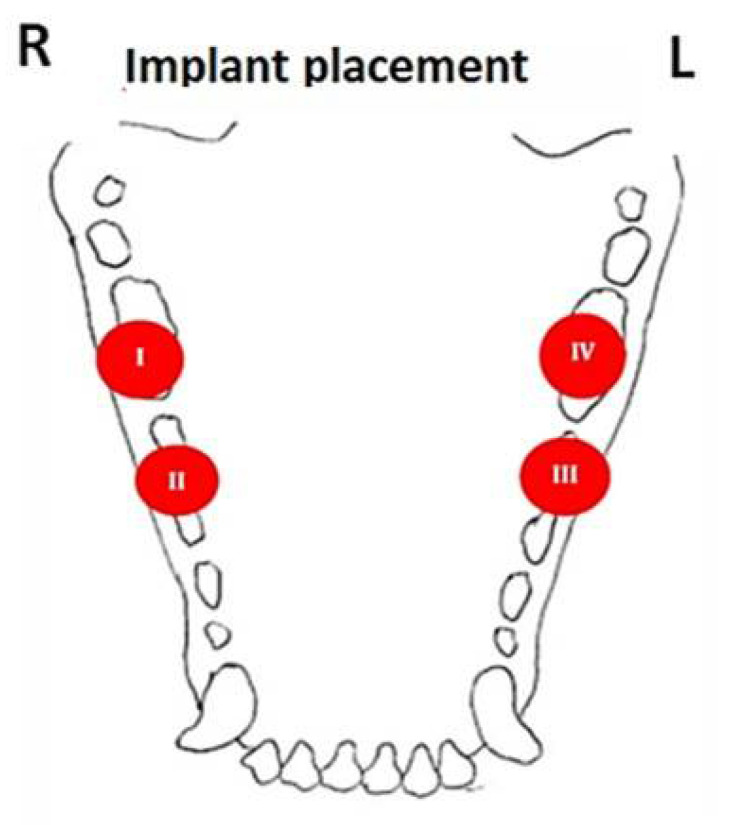
Schematic description of implant placement sites.

A total of 28 implants were placed: two on each side of the mandible (Figure 4). All implants were sand-blasted/acid-etched and of similar dimensions (10 mm long, 4.2 mm diameter). These were divided into twenty-one non-screw-type removable collar implants (experimental) and seven standard screw-type collars (control). The same modified drilling protocol was used for all implants.

To assess location (anterior vs. posterior mandible) impact, implants were placed as follows: group I—posterior mandible right—non-screw-type collar implants and group II—anterior mandible right- non-screw-type collar implants. To assess collar effect on the outcome, Group III—screw-type collar implants were placed at the anterior left side of the mandible (control), while Group IV—non-screw-type collar implants (experimental) were placed at the left side of the posterior mandible. The following parameters were measured and recorded: insertion torque, soft tissue healing, early implant failure, and EMBL at 2nd-stage surgery.

#### 2.3.3. Surgery

Extractions (Day 0): All dogs presented with a healthy dentition and periodontium before tooth extraction. Dental cleaning using an ultrasonic device was performed, followed by the computerized tomography scanning of the mandible. Following local anesthesia (lidocaine 2%/adrenaline 1:100000 X 5.4cc), the extraction of both-sides mandibular third and fourth premolars and first molars (P3, P4, M1) of each animal was performed using an atraumatic surgical technique. On the following days, the dogs ate as usual.

Implant placement (Week 14): A mid-crestal incision and a facial releasing incision exposed the edentulous bone crest. Full-thickness mucoperiosteal flaps were elevated to expose the site of implantation. Debridement was carried out to completely denude the bony walls from connective tissue remnants. Implants (4.2 mm diameter, 10 mm length, either screw-type collar internal hex or 4.2 diameter non-screw-type collar internal hex implant) were inserted until the implant’s shoulder was level with the surrounding bony crest. When crestal irregularities existed, the bone was leveled with the implant’s shoulder using an Ochsenbein chisel. After placement, the insertion element was removed, and the implants were sealed with cover screws.

Due to some residual minor irregularities of the bony crest, the distance between the bone crest associated with the buccal shoulder of the implant was measured to the nearest 0.5 mm using a manual periodontal probe. Flaps were replaced by simple interrupted and/or mattress lock sutures using 3-0 Vicryl Rapide™ (Johnson & Johnson, New Brunswick, NJ, USA).

#### 2.3.4. Insertion Torque

Implants were inserted into the mandibular bone. After osteotomy preparation, the implant was removed from the package and inserted into the osteotomy using the motor system by gradually increasing the insertion torque, starting at 25 N/Cm, every time implant could not be inserted anymore, until implant reached full insertion. All implants were placed using this protocol. After implantation, the insertion element was removed and the implants were visually examined for any problems or deviations from the expected results.

#### 2.3.5. Soft Tissue Healing

At week 28 (14 weeks post-surgery), mucosa wound healing was clinically classified as previously described by Tal 1999 [25]:

Class 0: The mucosa covering the implant is intact.

**Figure 5:**
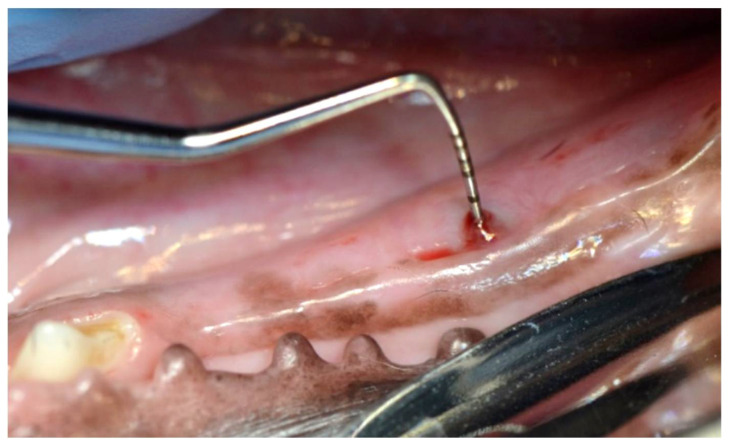
Oral implant communication can be detected with a periodontal probe, but the implant surface cannot be observed.

Class 1: A breach in the mucosa covering the implant is observed. Oral implant communication can be detected with a periodontal probe, but the implant surface cannot be observed without mechanically interfering with the mucosa (Figure 5).

Class 2: The mucosa above the cover screw is fenestrated; the cover screw is visible. The borders of the perforation’s aperture do not reach or overlap the boundaries of the cover screw at any point.

Class 3: The cover screw is visible. In some parts, the borders of the perforation aperture overlap the boundaries of the cover screw.

**Figure 6:**
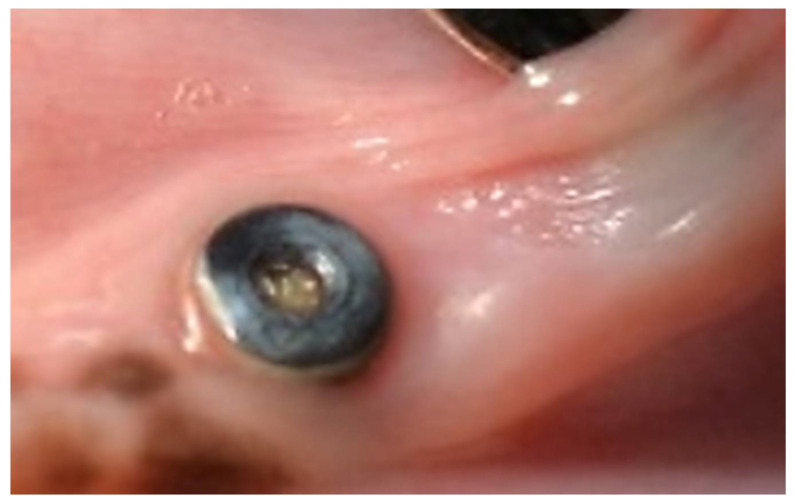
The cover screw is completely exposed.

Class 4: The cover screw is completely exposed (Figure 6).

#### 2.3.6. Early Bone Loss

At 14 weeks post-surgery, all implants were exposed, to assess their bone level. For implant exposure, the mucosa was incised and reflected as previously described [25,26,27,28]. Remnants of soft tissue were removed from the bony crest surrounding the implants, and measurements of the crestal bone level relative to the implant shoulders were repeated like the way it was performed at the time of implant placement. The buccal aspects of crestal bone level relative to the shoulder of each implant were measured to the nearest 0.5 mm using a hand periodontal probe marked from 1 to 10 mm. Data was collected (maximum MBL noted) for all implants and tabulated.

### 2.4. Statistical Analysis

Due to the study’s experimental (dogs) in vivo nature, a minimal animal sample size was used. The statistical analysis was performed using SPSS software version 24.0 (SPSS Inc., Chicago, IL, USA). One-way ANOVA (analysis of variance) with posthoc Tukey HSD (honestly significant difference) was used to compare between groups. A *p*-value of less than 0.1 was considered statistically significant.

## 3. Results

### 3.1. In Vitro Study on Stability

The pilot implants (n = 15) were inserted into the polyurethane block using the same drilling-and-insertion technique. Implants were fully inserted to the block level with insertion torque of 50 N/cm. It was concluded that the modified standard drilling sequence (final drill 4.2 diameter, up to 6 mm depth) for 13 mm length and 4.2 mm diameter implants allowed full non-screw-type collar implant insertion with high initial stability. 

### 3.2. Experimental Prospective In Vivo Study

#### 3.2.1. In Vivo Insertion Torque

All 28 implants were inserted successfully; implants were placed to the level of the crestal bone. The stability of 26 implants reached 55 N/cm, while the stability of 2 non-screw-type collar implants reached 40 N/cm (Table 1). There were no statistically significant differences between all 4 implant groups.

#### 3.2.2. Soft Tissue Healing

Out of 28 implants, soft tissue healing was classified as 0 in 19; 1 in 5; 2 in one implant; and 4 in 2 implants (Table 2). The average score of the controls (7 implants; group III) was 1.43 vs. 014 in the study group (group IV). This difference was statistically significant (*p* = 0.091). There was no statistically significant difference between group I and group II, i.e., no difference between anteriorly and posteriorly placed implants.

#### 3.2.3. Early Bone Loss

**Figure 7:**
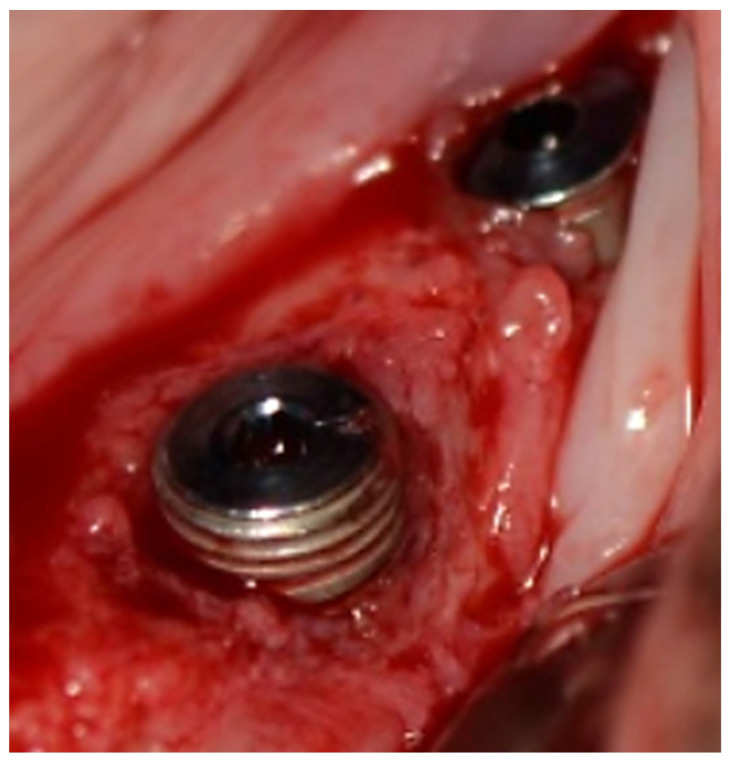
3 mm buccal bone loss.

Twenty-five implants showed no bone loss. Three implants in the control group (group III—standard screw-type collars.) demonstrated bone loss measuring 1, 2, and 3 mm (Figure 7). Their soft tissue classification was 1, 4, and 4, respectively. Test implants (Group IV) presented no bone loss in accordance with good soft tissue healing in 6 cases (class 0) and 1 with class 1 compromised healing not reflected in EMBL. The mean early bone loss in the control group III (screw-type collar) was 0.86 mm vs. 0 mm in the other three groups (non-screw-type collar) (Table 3). The difference was statistically significant (*p* = 0.087). 

## 4. Discussion

In the present study, implants covered with intact mucosa (Tal Class 0) presented no significant bone loss. On the other hand, implants with early spontaneous exposure (class 2–4) presented significant bone loss at the time of full exposure. This was expected based on previous clinical findings [25,26,27,28]. However, in the present study, EMBL was noted around screw-type collar implants only. EMBL reached up to 3 mm, and it was evident even in implants with class I soft tissue perforations (dog No. 3). Non-screw-type collar implants had no early bone loss even though implants were associated with jeopardized soft tissue healing. It may be speculated that non-screw-type collar implants suffered less from plaque retention and minimized bone pressure by using the modified drilling protocol, protecting the bony crest between stage I and stage II surgery, despite the early spontaneous soft tissue exposure.

Peri-implant health is linked, among other factors, with implant design [29,30]. Implant threads are intended to promote primary stability, increase total-bone-to-implant-surface contact area and improve stress distribution [31,32]. Using a non-screw-type collar in the newly designed implants could have jeopardized the initial primary stability. The purpose of the in vitro pre-study experiment was to evaluate whether the change introduced in the drilling protocol for non-screw-type collar implants, i.e.,—the use of a final drill of 4.2 diameter, for 6mm depth—did not jeopardize initial stability. Rigid foam blocks are accepted as an alternative material to human cadaver bone for testing and demonstrating new orthopedic implants, instruments, and instrumentation [33]. Considering drilling, 30 PCF rigid polyurethane foam is comparable to a human rib. This closed-cell foam conforms to ASTM F-1839-08 “Standard Specification for Rigid Polyurethane Foam for Use as a Standard Material for Testing Orthopedic Devices and Instruments” [33]. The in vitro test results demonstrated that, at 50 N/cm, primary stability is achieved for all implants tested. The results obtained in stage I surgery of the experimental in vivo study confirmed the ability to reach initial stability (40–55 N/cm). Initial stability was not compromised in both in vitro and in vivo experiments, alleviating the concerns of modifying the drilling protocol.

Different factors may result in the formation of the spontaneous early perforation of the mucosa covering the implants. The main and most common one is mechanical trauma to the mucosa over the implants, followed by tension in the tissue flaps. Bone debris produced during the osteotomy may be an additional predisposing factor. These were shown to sequestrate and become associated by chronic inflammatory cell infiltration as well as epithelial-covering reaction [25,26,27,28]. In the present study, non-screw-type collar implants presented with less spontaneous early perforations. It may be speculated that the use of a non-screw-type collar resulted in reduced bone pressure and yielded less bony sequestrate and accompanying chronic inflammation, leading to an improved epithelial covering. Moreover, it has been previously claimed that a screw-type collar implant model displayed 29% greater stress at the crestal bone compared with a non-screw-type collar implant model [34].

The longevity of dental implants relies upon the ability of peri-implant bone to withstand applied forces [34,35]. The application of exaggerated stress may cause bone resorption [36,37,38,39].

A review that compared the role of dental implant collar characteristics on maintaining peri-implant health concluded that machined collar implants demonstrated higher marginal bone loss vs. rough-surface ones [40]. Thread modifications (micro threads and LASER micro texturing) did not reduce bone resorption [40]. Those results are compatible with the present study results, which demonstrated that screw-type collars did not perform better than non-screw type collars as long as rough-surface implants were used.

A thickness of approximately 2 mm of the buccal bone was associated with minimal EMBL [41] and less peri-implant mucosal recession [42].

Regarding the bone quality, it was shown that type-4 bone presented minimal EMBL [41]. Contradictorily, a review article found no minimum buccal bone thickness that will completely avoid peri-implant bone loss and maintain soft tissue stability after implant placement [43]. Those reports motivated us to explore the impact of implant location more precisely. Increased bone thickness in the posterior mandible with lower bone quality compared to the anterior sites might have influenced the results of EMBL. According to the present findings, the collar design is important, while the impact of implant location is still questionable. Such results are in agreement with the latter reports [41,42,43]. 

Strength: The assessment of EMBL changes is critical in the short- and long-term evaluations of peri-implant disease in implant dentistry. Information obtained from intra-oral radiographs is confined to the mesio-distal aspects of dental implants. Cone-beam computed tomography (CBCT) may provide radiographic images from circumferential aspects. However, clinical evaluation will give the best results. This is an in vivo animal study in the dog that enabled precise follow-up of the "clinical" tissue changes under strict clinical conditions in a GLP laboratory. 

Limitations: A review [44] of 133 included studies regarding peri-implant disease demonstrated that dogs were the most commonly used animal, followed by monkeys, mice, micro-/mini-pigs, and rats. For this reason, dogs were selected for the present study. A significant drawback to the use is that they display more bone loss than monkeys or smaller animals. The time required to achieve a certain amount of bone loss also varies in dogs or monkeys. Once preliminary results are obtained (the present study), a similar study should be performed on human patients for a better clinical outcome. Although beagle dogs provide an excellent animal model, before final conclusions be made, it is suggested that a pilot human study be undertaken.

Not all patients requiring dental implants are males. The minimum number of animals in accordance with current regulations and scientific integrity was used. Female dogs were not considered for this study due to the small number (7) of animals allowed for the study. Using both genders would have made statistical analysis impossible. In the future, human studies should asses gender differences regarding EMBL.

## 5. Conclusions

In view of the clinical implications that spontaneous early exposure may influence peri-implant health, it may be suggested that rough-surface, non-screw-type collar implants show promising results in minimizing early bone loss. Future human studies are required to elucidate the role of rough-surface, non-screw-type collar dental implants in maintaining the longevity of peri-implant health.

## Figures and Tables

**Table 1:** Insertion torque (N/cm).

Dog No.	Implant No.			
	Group I	Group II	Group III	Group IV
1	55	55	55	55
2	55	55	55	55
3	55	55	55	55
4	55	55	55	55
5	40	40	55	55
6	55	55	55	55
7	55	55	55	55
Average	52.86	52.86	55.00	55.00
	5.67	5.67	0	0
*p*	NS		NS	

**Table 2:** Scoring according to Tal soft tissue classification [20,21,22,23].

Dog No.	Implant No.			
	Group I	Group II	Group III	Group IV
1	0	0	0	0
2	2	4	4	0
3	0	0	1	0
4	0	0	0	0
5	0	0	4	0
6	1	1	1	1
7	0	0	0	0
Average	0.43	0.71	1.43	0.14
SD	0.79	1.50	1.81	0.38
*p*	NS		0.091	
	**Group III**	**Group IV**	**Total**	
N	7	7	14	
∑χ	10	1	11	
Mean	1.4286	0.1429	0.786	
∑χ^2^	34	1	35	
Std.Dev	1.8127	0.378	1.4239	
	*SS*	*df*	*MS*	
Between treatments	5.7857	1	5.7857	
Within treatments	20.5714	12	1.7143	*F* = 3.375
Total	26.3571	13		

The *f*-ratio value is 3.375. The *p*-value is 0.091071. The result is significant at *p* < 0.10.

**Table 3:** Buccal EMBL at stage-II surgery (mm).

No.	Implant No.			
	I	II	III	IV
1	0	0	0	0
2	0	0	2	0
3	0	0	3	0
4	0	0	0	0
5	0	0	1	0
6	0	0	0	0
7	0	0	0	0
Average	0	0	0.86	0
SD	0	0	1.21	0
*p*	NS		0.087	
	**Group 1**	**Group 2**	**Total**	
N	7	7	14	
∑χ	b6	0	6	
Mean	0.8571	0	0.429	
∑χ^2^	14	0	14	
Std.Dev	1.215	0	0.9376	
	*SS*	*df*	*MS*	
Between treatments	2.5714	1	2.5714	
Within treatments	8.8571	12	0.7381	*F* = 3.48387
Total	11.4286	13	0.429	

The *f*-ratio value is 3.48387. The *p*-value is 0.086587. The result is significant at *p* < 0.10.

## Data Availability

Data supporting reported results can be found in the table.
